# Use of strontium isotope ratios in geolocation of Guatemalan population: Potential role in identification of remains

**DOI:** 10.1111/1556-4029.15116

**Published:** 2022-08-09

**Authors:** Ryan Austin, Gillian Fowler, Jonathan J. Cooper, Marco Perez Tanchez, Ruth Croxton, Jane Evans, David F. Thompson

**Affiliations:** ^1^ School of Arts, Humanities and Social Sciences, Plymouth Marjon University Plymouth UK; ^2^ School of Chemical and Physical Sciences, Keele University Keele UK; ^3^ School of Life Sciences, Lincoln University, Joseph Banks Laboratories Lincoln UK; ^4^ Department of Applied Sciences Northumbria University Newcastle upon Tyne UK; ^5^ Natural Environmental Isotope Facility British Geological Survey Nottingham UK

**Keywords:** forensic anthropology, geolocation, Guatemala, hair, human provenancing, region of origin, strontium isotopes, thermal ionization mass spectrometry, TIMS

## Abstract

Within post‐conflict communities, attempts to identify and repatriate unidentified and missing individuals poses a difficult task. As current forensic strategies commonly lack the capacity to provide region of origin assessments, forensic anthropologists/investigators are often unable to identify sources of DNA for kinship analysis. Using Thermal Ionization Mass Spectrometry (TIMS), hair samples from 10 volunteers were used to assess the variation in strontium isotope ratios (^87^Sr/^86^Sr) between extant people in Guatemala City and Coban; with a leach (external) and digest (dietary) signal analyzed for each sample. A two‐way anova demonstrated that the difference between ^87^Sr/^86^Sr of Guatemala City and Coban was statistically significant (*F* [1, 16] = 259.839, *p* < 0.05), with no statistically significant differences observed between leach and digest ^87^Sr/^86^Sr (*F* [1,16] = 4.319, *p* = 0.054). Overall, individuals from Coban demonstrate ^87^Sr/^86^Sr comparable to previously recorded baseline values, demonstrating a minimal change in diet which is reflected in associated surveys. Volunteers from Guatemala City, however, show a marked shift in ^87^Sr/^86^Sr away from predicted values highlighting the potential influence of imported goods. The results here highlight the applicability of ^87^Sr/^86^Sr in hair to serve as a potential tool to support the identification of unknown individuals in Guatemala in a forensic context.

**Highlights**

- ^87^Sr/^86^Sr from the hair of living individuals from Guatemala were analyzed and compared.
- For each sample, a leach (external) and digest (internal) ^87^Sr/^86^Sr signal was collected.
- Guatemala City and Coban could be distinguished based on ^87^Sr/^86^Sr.
- Presence of elevated ^87^Sr/^86^Sr in Guatemala City suggests the influence of non‐local food.
- Caution when using modern ^87^Sr/^86^Sr for provenancing remains from Guatemalan internal conflict.

## 1 INTRODUCTION

The discourse of identification and repatriation in Guatemala has been a reoccurring theme for the country's forensic expertise, due to a history of the displacement of individuals through migration or enforced disappearances. This trend has been observed since the 1960s whereby Guatemala's internal conflict, fought until 1996 between governmental forces and guerrilla groups mainly drawn from indigenous populations, saw approximately 500,000–1.5 million individuals fleeing the country or internally displaced. Despite estimates that 200,000 people were killed or disappeared, of those identified, the Mayan population represents more than 80% of documented victims with the Ladino population (those of mixed European and indigenous heritage) making up ~17%, who have become collectively known as ‘Los Desaparecidos’ or ‘The Missing’ [1, 2, 3].

Today, Guatemala's modern ‘Missing’ are characterized predominantly by individuals who disappeared as a result of migration efforts, spurred by political and socioeconomic marginalization with individuals often seeking refuge, by migrating to the United States. Despite individuals from Guatemala (in addition to El Salvador and Honduras) making up more than 52% of apprehensions at the US‐Mexico border [4], many individuals do not reach their intended destination, as a result of criminal interference or through succumbing to the harsh environment during their journey. When the remains of ‘Missing’ individuals are found, forensic intervention is required to help provide an identification.

Forensic strategy dictates that identification efforts are focused on the utilization of DNA. However, the use of DNA relies on collecting appropriate familial reference samples, whereas region of origin assessments can prove pivotal in both focusing DNA efforts and excluding potential alternative identities. In Guatemala, while region of origin assessments may be possible by visual assessment of traditional indigenous clothing that is distinct amongst different regions within the country [5], such assessment is subjective and the success and indeed presence of such artifacts relies heavily on burial conditions [6]. The use of region of origin assessments not only serves as a strategy to stratify potential identities and to focus DNA efforts but also has a cultural significance, with proper burial ensuring a restful transition into the afterlife in accordance with traditional Mayan beliefs [7].

Isotopes offer the potential to track the migration of individuals, through tissues such as hair, teeth, and bone, by representing region of origin at various stages during life. In particular, the strontium isotope ratio ^87^Sr/^86^Sr is used frequently in geolocation studies as it differs geographically due to heterogeneity in bedrock geology and atmospherically derived sources [8]. These environmentally controlled isotopes are readily available in the body's hard tissues where the resulting strontium isotope ratio represents an accumulation of the ratios from areas where dietary consumables are obtained [9, 10]. In the body, intestinal absorption of strontium results in the manifestation of strontium in the body's hard tissues (ie bones and teeth) where it readily substitutes for calcium in the apatite of both structures [11, 12]. Teeth, in particular the enamel, provides region of origin ^87^Sr/^86^Sr during childhood, with the first permanent molar often chosen for ^87^Sr/^86^Sr analysis, where the mineral formation, and therefore strontium incorporation into the enamel of that tissue, ceases approximately within the first 3.5 years of childhood [13]. Alternatively, bone samples such as femoral cortical bone, through remodeling, reflect the last 10–15 years of life [14]. While the comparison of ^87^Sr/^86^Sr in bone and teeth offers the ability to identify migration from childhood residency, the inability to remodel and resistance to diagenesis means tooth enamel is the preferred tissue for region of origin assessments [15].

Ideally teeth would be the focus for sample collection and tissue of choice when analyzing the remains of missing individuals. However, sampling an extant population is not often feasible, due to its invasive nature and issues regarding donation, particularly in countries such as Guatemala, where Mayan regions often rely on traditional ‘teeth pullers’ [16] or international teams/volunteers working remotely to provide dental care [17], rather than a central dental practice. In this scenario, hair can serve as a proxy to identify regional differences in ^87^Sr/^86^Sr from human populations.

Unlike strontium in bone and teeth, which is found within the crystalline hydroxyapatite structure, strontium ions (Sr^2+^) in hair are bound as a result of the negative charge associated with peptides through ionic bonds [18]. As a tissue which exists externally, hair is influenced by strontium from two sources firstly, an internal source, the ^87^Sr/^86^Sr in blood where trace metal exchange occurs until subcutaneous formation prior to exposure from the scalp [19]. Secondly, it is influenced by external strontium sources from the environment, such as bathing water, aerosols and dust [20]. Strontium is found particularly in the internal portion of hair, such as the cortex and medulla but also in the external structures, such as the cuticle, where metals, such as calcium, adhere to the assumption that the chemical similarity between strontium and calcium is the cause [21]. The exogenous ^87^Sr/^86^Sr influence on hair is demonstrated through an increasing concentration in ^87^Sr/^86^Sr from the proximal to distal portions and through transverse cross sections [19], highlighting the permeability of hair as a result of the narrow overlap between cells in the cuticle layer [22].

Hair provides a more recent indicator of provenance or travel history, with 1 month of residency represented by ~1 cm of hair growth [23]. Such physiological factors allow researchers to establish spatial and temporal information through the sequential analysis of hair segments from the tip to the root, tracking migration [24]. Alternatively, bulk analysis of hair is often used in studies when such distinction is non‐essential and provides an accumulative ^87^Sr/^86^Sr signal [19, 25, 26]. The use of hair provides a number of additional benefits in the determination of bioavailable strontium from an extant population, in that it is readily available, collection/sampling is non‐invasive, and it offers reportedly, the potential to discern between environmental and dietary sources of strontium by leaching and digesting the hair in a suitable acid and analyzing the resulting (leachate and digest) solutions separately [19]. With strontium isotopes demonstrating a lack of fractionation, unlike light isotopes such as oxygen, hydrogen, nitrogen and carbon [8], comparative strontium isotope ratios should be reflected in the hair, teeth and bone of a sedentary individual. Despite this, there is a consensus that samples analyzed for forensic purposes should be compared to isotopic reference values of the same type, ie, bone to bone, teeth to teeth [27]. However, understandably unlike hair, such samples are often unobtainable or impractical to collect [27].

Much of the current understanding on bioavailable strontium in Guatemala is based on archeological populations [28, 29, 30, 31, 32, 33, 34, 35]. With self‐sufficient populations such as the Maya, dietary strontium, and subsequently bioavailable strontium in the community, would be expected to reflect the ^87^Sr/^86^Sr ratios of the underlying geology in which they reside. However, dietary preferences and the availability of imported goods can alter the expected bioavailable ^87^Sr/^86^Sr outside those reported in their archeological ancestors [25, 36]. As a result, for this study, the use of modern hair samples for the geolocation of a more contemporary population was chosen given the complexity of the modern globalized diet.

This study therefore represented an exploration of the potential to use hair samples as a means of geolocation. Two populations were chosen for this study with distinct underlying geology, which may in turn be reflected in strontium isotope intake through consumption of locally grown produce.

### 1.1 Geology of mesoamerica

**FIGURE 1:**
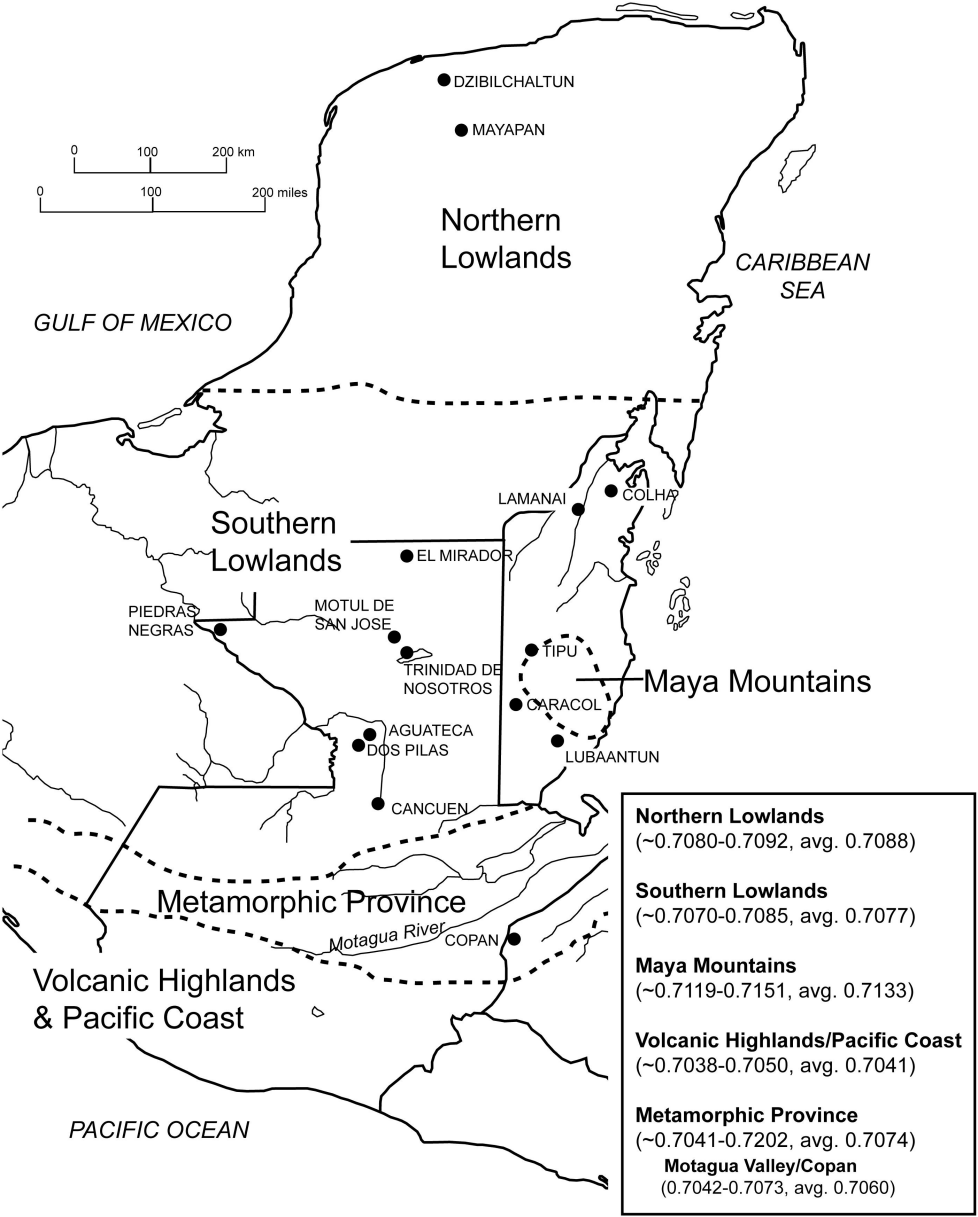
A map of the Maya region from Thornton [35] adapted from Hodell et al with associated data [28] (Elsevier©), Guatemala is highlighted using a bold outline from the southern lowlands to the apex of the Pacific coast. Reproduced with permission from Elsevier [28, 35]. Copyright 2003, 2011, Elsevier.

When characterizing ^87^Sr/^86^Sr in Guatemala, Hodell et al is often used as the reference [28] with his research into the Mayan regions in Mesoamerica forming the basis for comparison throughout works from the region [29, 30, 31, 32, 33, 34, 35]. Overall, the Mayan regions can be dissected into six regions: the Lowlands (Northern and Southern), the Metamorphic Province, Volcanic Highlands, Pacific Coast and Maya Mountains (Figure 1).

Of these areas, two exist outside of Guatemala with the Northern Lowlands and Maya Mountains found in Mexico and Belize, respectively. The Southern Maya Lowlands are found in the north of Guatemala and incorporate the departments of Petén and Alta Verapaz [37, 38]. This region founded upon marine carbonates such as limestone exhibits an ^87^Sr/^86^Sr range of 0.7070–0.7085, with values decreasing as a function of proximity to the coast, due to increasing ^87^Sr/^86^Sr of seawater [31, 35]. The Metamorphic Province is found between the Southern Lowlands and Volcanic Highlands, with ^87^Sr/^86^Sr values (0.7041–0.7202) reflecting the diversity of exposed rock types including Paleozoic meta‐sediments, serpentinites, and granites, with the Mortagua Valley existing as a sub‐region which extends into nearby Copan, Honduras [28]. The Volcanic Highlands and Pacific Coast are found in the south of country and while separate geographically, both areas are characterized by geological young deposits with the Quaternary Alluvium belt of the Pacific Coast resulting from the erosion of tertiary and quaternary volcanics of the highlands. Resultantly, both regions exhibit similar ^87^Sr/^86^Sr ratios (0.7038–0.7050) the lowest in the country [28, 31, 35].

## 2 MATERIALS AND METHODS

### 2.1 Materials

Through established contacts in both locations, ten hair samples were provided by Guatemalan volunteers, with 5 samples from Coban and 5 from Guatemala City found in the Southern Lowlands and Volcanic Highlands, respectively. The sample collection was facilitated by a translator who also acted as a gatekeeper when working specifically within the indigenous community. This individual was trusted by the community with previous experience working within forensic exhumations.

Informed consent was obtained, and individuals were provided with an information leaflet in Spanish which outlined the study's background, aims, methodology and publication examples before the samples were collected. Volunteers were then asked to answer a series of questions of isotopic significance as part of a lifestyle questionnaire. The integrity, continuity and reliability of each hair sample were ensured through the collection of the hair from each volunteer from the same site at the back of the head using sterile scissors. The hair sample was then wrapped in aluminum foil and placed in a labeled sample bag before being attached to the associated volunteer's isotope questionnaire. The corresponding information collected for each sample through the isotopic questionnaire is presented in Table 1.

**TABLE 1:** Details of isotopic interest from individuals whose samples were analyzed for ^87^Sr/^86^Sr

Sample name	Sex	Age (years)	Area of residence	Mobility habits for work	Water consumed	Water used in home	Water origin	Origin of food	Hair dyed
GUAC‐2/17	F	13	Guatemala City	None	Bottled	Piped Public Service	Municipal	Local	No
GUAC‐4/17	F	34	Guatemala City	None	Bottled	Piped Public Service	Municipal	Local/unknown	No
GUAC‐7/17	F	N/A	Guatemala City	None	Filtered	Piped Public Service	Municipal	Local	No
GUAC‐11/17	M	N/A	Guatemala City	Home to Work	Spring	Water Well	Municipal	Local	No
GUAC‐12/17	F	N/A	Guatemala City	Home to Work	Stream	Piped Public Service/Water Well	Municipal	Local	No
COB‐2/17	M	48	Carcha	Coban for Study	Purified filtered	Piped Public Service	Municipal	Answer not provided	No
COB‐3/17	M	37	Coban	Travel to Senachu	Water well/piped public service	Piped Public Service	Municipal	Local	No
COB‐9/17	M	28	San Cristobal Verapaz	Coban for Study	Stream/bottled	Piped Public Service	Municipal	Answer not provided	No
COB‐10/17	F	55	San Cristobal Verapaz	Coban for Study	Stream/bottled	Piped Public Service	Spring of Chiculha	Answer not provided	No
COB‐14/17	M	45	Aldea Pasmolón Tactic	Coban for Study	Bottled	Piped Public Service	Municipal	Answer not provided	No

### 2.2 Analytical methods

Upon returning from Guatemala, samples were stored in a secure unit at the Analytical Suite in the Lennard‐Jones building at Keele University for 3 months. They were subsequently transferred to the Isotope Geoscience Facilities at the British Geological Survey for analysis.

To discriminate between exogenous (surficial) and endogenous (structurally bound) strontium, the samples were first leached in hydrochloric acid (HCl) to remove surficial contamination (Tipple et al, method 3) and the cleaned hair was then digested in nitric acid (HNO_3_) to release the structurally bound strontium [19].

### 2.3 Hair preparation – removal of surficial strontium using acid leach

Hair was removed from the aluminum wrap with a Milty Zerostat 3 antistatic gun (Armor Home) in order to dissipate the static charge from loose hair found in the bag. The hair samples were transferred to a Class 100 Laminar flow hood where they were placed in a pre‐cleaned Savillex© beaker and 7 ml of Teflon© distilled 0.1 M HCl acid were added. The samples were left for 20 min. The eluent was then transferred to a new sterile test tube. The hair sample was washed with 1 ml of Milli‐Q (18.2 Ωcm) water, which was then added to the leaching acid in the test tube. This wash step was completed three times, with each leach solution examined visually to confirm no residual hair was present. The combined acid leachate and water washes were centrifuged to remove any particulate material, and the supernatant fluid was decanted into a cleaned Savillex© beaker and evaporated to dryness at 100°C.

### 2.4 Hair preparation – digest

The cleaned hair was digested using 10 ml of Teflon distilled 8 M HNO_3_ on a hotplate at 100°C and allowed to evaporate to dryness. The sample was converted to chloride form by adding 5 ml of 6 M HCl and evaporating to dryness.

Once dissolved and taken up in calibrated 2.5 M HCl, the Sr was separated from both the leachate and the hair samples using an Eichrom© AG50‐X8 cation resin. The strontium isotope composition was determined by Thermal Ionization Mass Spectrometry (TIMS) using a Thermo Triton© multi‐collector mass spectrometer. Samples were loaded on to single rhenium filaments using TaF activator following the method of Birck (1986) [39]. Samples were run in peak jumping mode for 100 scans and to an internal precision of ≤0.00001 (2 SE). Strontium procedural blanks were ~50 pg. The reproducibility of the international standard NBS987 was ±0.00006 (2SD, *n* = 46) over the period of analysis. All data were corrected to an NBS 987 standard ^87^Sr/^86^Sr value of 0.710250.

### 2.5 Statistical analysis

Statistical analysis was completed using SPSS v25. Data were tested for conformity of requirements of parametric analysis prior to use of two‐way anova to assess the effects of location, nature of sample and their interaction.

## 3 RESULTS

**FIGURE 2:**
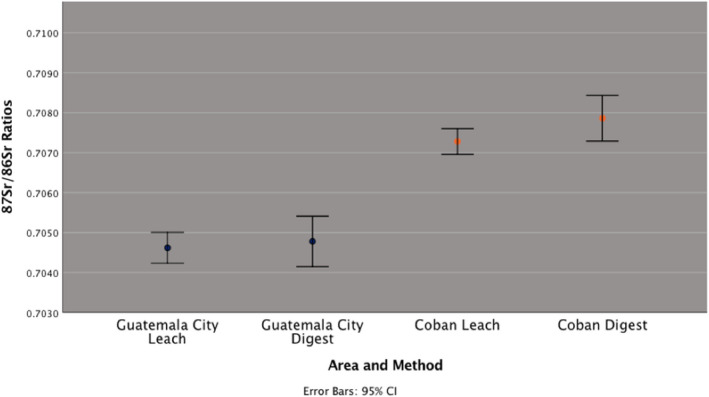
Mean ^87^Sr/^86^Sr ratio (±SD) by location and method

When assessing the data in Table 2, it can be seen that individuals from Coban exhibit comparatively higher ^87^Sr/^86^Sr in leach (0.7069–0.7076, mean = 0.7073 ± 0.0003) and digest (0.7072–0.7085, mean = 0.7079 ± 0.0005) than leach (0.7043–0.7050, mean = 0.7046 ± 0.0003) and digest ^87^Sr/^86^Sr (0.7043–0.7055, mean = 0.7048 ± 0.0005) from inhabitants of Guatemala City (Figure 2).

**TABLE 2:** Strontium isotope ratios of corresponding leachate and digest values using hair collected from Guatemala (GUAC‐Guatemala City/COB‐Coban) analyzed at the NERC Laboratory, Keyworth, UK

Sample name	Sample preparation	^87^Sr/^86^Sr	Mean (SD)
GUAC‐2/17	Leachate	0.7043	0.7046 (0.0003)
GUAC‐4/17	Leachate	0.7047	
GUAC‐7/17	Leachate	0.7050	
GUAC‐11/17	Leachate	0.7048	
GUAC‐12/17	Leachate	0.7043	
COB‐2/17	Leachate	0.7069	0.7073 (0.0003)
COB‐3/17	Leachate	0.7073	
COB‐9/17	Leachate	0.7072	
COB‐10/17	Leachate	0.7076	
COB‐14/17	Leachate	0.7074	
GUAC‐2/17	Digest	0.7043	0.7048 (0.0005)
GUAC‐4/17	Digest	0.7055	
GUAC‐7/17	Digest	0.7048	
GUAC‐11/17	Digest	0.7050	
GUAC‐12/17	Digest	0.7043	
COB‐2/17	Digest	0.7079	0.7079 (0.0005)
COB‐3/17	Digest	0.7078	
COB‐9/17	Digest	0.7072	
COB‐10/17	Digest	0.7085	
COB‐14/17	Digest	0.7079	

From the ratios provided in Table 2, a two‐way anova demonstrated that there was a statistically significant difference observed between the ^87^Sr/^86^Sr from both locations *F* (1, 16) = 259.839, *p* < 0.05. Conversely, when comparing leach and digest ^87^Sr/^86^Sr no statistically significant differences were found between the two sample‐types *F* (1, 16) = 4.319, *p* = 0.054, nor was the interaction between location and method, *F* (1, 16) = 0.255. Despite a statistically significant difference between both locations, the sample size is limited and would benefit from additional samples to confirm such differences.

## 4 DISCUSSION

Overall, this research has focused on ten hair samples from Guatemala City and Coban with five samples collected from each location. Using ^87^Sr/^86^Sr, Guatemala City and Coban are distinguishable (*p* < 0.05); however, crossover exists between leach and digest values for the same location (*p* < 0.054), which is reflected in Figure 2. It is important to note that whilst the statistical analysis here demonstrates the potential of how strontium isotopes can be used and exploited, the statistical analysis is based on a limited dataset, with a minimum of 30 samples often suggested [15]. Although such differences in ^87^Sr/^86^Sr may seem negligible numerically, within the context of the instrumental capabilities, such differences are large isotopically, with the TIMS instrument demonstrating a precision of ±0.000006 for strontium in this study [15].

The ability to distinguish between Coban and Guatemala City on the basis of ^87^Sr/^86^Sr would be expected given the underlying geology. In terms of consumables, whilst heterogeneity existed in the water consumed during the day, the majority of respondents in both populations confirmed that municipal water was utilized in the home.

However, contrary to expectations, the majority of individuals from Guatemala City recorded that locally grown food was consumed (100%), when compared to Coban where the majority of individuals were unable to confirm if local food was consumed (with bioavailable ^87^Sr/^86^Sr in individuals influenced, as a key factor, by the origin of foodstuffs) [40].

Based on the values provided by Hodell et al, individuals from Coban as part of the Alta Verapaz region in the Southern Lowlands, consuming a diet predominantly consisting of local produce, would be expected to exhibit an internal ^87^Sr/^86^Sr spanning from 0.7070–0.7085 [28] with the city founded on Cretaceous limestone deposits. Guatemala City located within the traditional Southern Highlands, founded on Tertiary and Quaternary volcanic deposits, would be expected to exhibit an ^87^Sr/^86^Sr range between 0.7038 and 0.7050 [28]. Overall, when the leach and digest ^87^Sr/^86^Sr for Coban are considered, the results gained in this study fall within the range offered in the literature [28, 29, 30, 31, 32, 33, 34, 35]. Similarly, whilst the ^87^Sr/^86^Sr of the leach portion for Guatemala City is represented by previously reported values, the digest values within this study exhibit a comparatively higher ^87^Sr/^86^Sr of 0.7043–0.7055. This study highlights that values gained for bedrock, water, soil, and plants previously are useful for the characterization of the external (leach) ^87^Sr/^86^Sr (dictated by the origin of water used to wash the hair), of both regions. However, in regards to digest ^87^Sr/^86^Sr, while the ratios of four out of five samples from Guatemala City in this study coincide with the range reported in previous studies [28, 29, 30, 31, 32, 33, 34, 35] the ratio exhibited by GUAC‐4/17 falls outside of such baseline values. Through analyzing the isotopic questionnaire provided by GUAC‐4/17 it was noted that the individual not only resided in the capital but also worked within the city, excluding the possibility of the data being skewed by possible migration [30]. Resultantly, while relative conformity exists between bioavailable ^87^Sr/^86^Sr in this dataset and geological baselines, it appears such methods could be less effective in determining the dietary ^87^Sr/^86^Sr available to individuals from Guatemala City.

Modern population data offer a potential explanation to this phenomenon. Overall, when assessing nutrition, studies have largely focused on factors such as population, increasing socioeconomic status and urbanity when assessing the gradual shift from a traditional diet. Whilst Coban and Guatemala City represent largely urbanized populations (99.8% and 100%, respectively) there is heterogeneity in the proportion of Mayan and Ladino residents that constitute the respective populations [41, 42]. Amalgamating research from the Institute of Nutrition of Central America and Panama (INCAP) and the Cross‐Cultural Research on the Nutrition of Older Subjects Study (CRONOS), Bermudez et al demonstrated that modern indigenous diets show 76% similarity to the diets eaten by individuals during the 1960, from foods which could be sourced locally [43]. Comparatively, it was found that only 72% of such foods contribute to the modern Ladino diet [43]. While the impact of population shows an interesting tendency, the capability to distinguish between Mayan and Ladino diets at the same location would offer greater clarity in assessing the impact of population for this study.

However, despite apparent population differences, the presence of elevated ^87^Sr/^86^Sr, away from locally defined ratios in Guatemala City, is most likely due to a combination of access to imported goods along with socio‐economic factors [44, 45]. The higher proportion of Ladino individuals in the capital, often characterized as more economically prosperous, resulted in supermarkets such as Walmart, who import 85% of produce, originating from within the countries' capital to grow exponentially from the 1980s (with the supermarket sector commanding 32% of Guatemala's food market as early as 2002) [44]. Importantly, therefore, whilst respondents from Guatemala stated that food was local in origin, it is likely that this was assumed based on geographical proximity to its area of purchase.

As the data collected in this study represent a modern population, the applicability of modern values to characterize ‘The Missing’ requires an understanding of the population involved during the internal conflict in relation to both diet and migratory habits. Whilst the internal migration of individuals within the department of Alta Verapaz (ie Coban, San Cristobal Verapaz, Carcha and Aldea Pasmolón Tactic), for seasonal work has occurred since the 19th century, by characterizing the contemporary indigenous diet of today as being 76% representative of that observed during the time of the internal conflict, demonstrates a change in dietary habits [44, 46].

In this assessment, indigenous communities are often only afforded access to small informal retailers, with the majority of foods either being sourced locally, via supplementation from subsistence farming or through markets [44, 47]. Although in regards to the latter, modern food networks increasingly mean food at such markets originate from across the country [48]. However, with a diet more closely representing that of individuals involved during the internal conflict and with bioavailable ^87^Sr/^86^Sr values that fall within those reported for traditional baseline materials, it would appear that the values presented in this paper would reflect the ^87^Sr/^86^Sr ratios for the population in Coban during the conflict. Conversely, inhabitants of Guatemala City, with a population characterized largely by individuals with higher income, are afforded access to a diet demonstrating a greater deviation from that consumed in the 1960s, reflecting in the higher ^87^Sr/^86^Sr range reported in this study when compared to previously gathered baseline data.

The findings presented here demonstrate an argument that the ^87^Sr/^86^Sr values for Coban during both periods have remained homogenous and the hierarchical trend of individuals residing in the Southern Lowlands reflecting higher ^87^Sr/^86^Sr than those in the Southern Highlands is still prevalent. However, the presence of a shift in modern bioavailable ^87^Sr/^86^Sr from baseline values in Guatemala City suggest that any comparisons made to individuals involved in the country's internal conflict should be used with caution. To establish whether this phenomenon is observed within the wider population, in order to create a more accurate representation of the population a larger and more randomized sample should be analyzed [15]. Additionally, in order to test this applicability of the values gained here to the population of the internal conflict, empirically, samples should be sought from individuals whose identity has already been established. However, this would require working closely with the indigenous communities and families for permission.

It is worth mentioning that whilst hair has been used to ascertain the bioavailable ^87^Sr/^86^Sr in the modern population, when analyzing the remains of ‘The Missing’ such values should be used as a baseline on which to compare to those gained from bone and teeth. This is due to concerns regarding the structural and taphonomic changes that occur in hair that results in the alteration of the original bioavailable ^87^Sr/^86^Sr [49]. To further clarify, for comparison to the remains of ‘The Missing’, the digest ratios presented here are of most importance. However, both leach and digest ^87^Sr/^86^Sr ratios may be used on the hair of living populations, or more recent remains that have not been subject to burial, with such conditions potentially causing degradation of the hair tissue [50].

Finally, to build upon the encouraging findings of this study, more work is required to enable the technique to be used across the field in Guatemala. Work is now being conducted by the authors in partnership with local forensic organizations to not only increase the number of samples (ie hair and water) but also their distribution across the country. To validate the use of strontium isotopes in the country, an analysis of skeletal remains (ie bone and teeth) of known origin should be conducted. Once verified the methods used could serve as a basis for developing standard operating procedures (SOPs) within the country. To our knowledge, Thermal Ionization Mass Spectrometry (TIMS) or alternative instruments such as Multi‐Collator Inductively Coupled Plasma Mass Spectrometry (MC‐ICP‐MS) are not widely available in the country and are not used for forensic purposes currently. Therefore, while in order to provide this capacity for routine domestic forensic investigations would rely on substantial funding, outside the current partnership, a short term solution would be to outsource a small number of samples, potentially those from individuals where there is very little material present which would help aid in identification.

## 5 CONCLUSION

Overall, this study has demonstrated that the use of ^87^Sr/^86^Sr in modern human hair has the potential to discern between areas within Guatemala by providing a comparison between two areas within the country, namely Guatemala City and Coban. Although the comparisons made here are centered on a modern population, based on the similar dietary and migratory patterns of Mayan individuals from 1960 to the present day, it is likely that areas incorporating such individuals are likely to be reflected by modern populations. However, with a difference observed between previously reported baseline values and the modern‐day samples presented here, it appears that individuals from Guatemala City are less likely to represent the population at the time of the internal conflict. To confirm this, future work should focus on increasing the sample size of modern individuals with samples from identified individuals sourced for comparison. Finally, although this study has utilized ^87^Sr/^86^Sr, future studies would benefit from the adoption of a multi‐isotope approach where researchers are demonstrating success in order to stratify areas which are founded on the same geological foundations [10, 31, 51].

## FUNDING INFORMATION

Funding provided through the University of Lincoln Joseph Banks PhD Scholarship.
